# Routine Radiologic Demonstration of Gall Stones

**Published:** 1916-02

**Authors:** 


**Routine Radiologic Demonstration of Gall Stones.** C. W. Lippman, San Francisco, Calif. State Jour. of Med., Dec., 1915. "To treat the stomach in gall bladder disease is like pouring water into the fire alarm box to put out the fire."—Mayo. Unlike many authorities, Lippman holds that, while gall stones constitute a surgical disease, cholecystitis calls for medical treatment—a view with which we heartily concur, with allowance for exceptions in either direction. Neither physical examination nor symptomatology afford a satisfactory basis for discrimination. Direct demonstration of calculi on plates is possible in 40-60% of cases. (This probably means that about this percentage consist of calcium-bilirubin calculi of sufficient size to cast a distinct shadow. Ed.) Indirect evidence of value in diagnosing gall bladder disease consists in (1) hepato-fixation of stomach; (2) high fixation of hepatic flexure; (3) distortion of duodenal cap; (4) tender point outside of duodenal shadow. Conversely, a shadow in the region of the gall bladder may mean (1) right renal calculus; (2) calcareous deposit in tuberculous right kidney; (3) calcareous deposit in rib cartilage; (4) calcareous lymph node; (5) enterolith.

